# Augmented Reality in Emergency Medicine: A Scoping Review

**DOI:** 10.2196/12368

**Published:** 2019-04-17

**Authors:** Brendan William Munzer, Mohammad Mairaj Khan, Barbara Shipman, Prashant Mahajan

**Affiliations:** 1 Department of Emergency Medicine University of Michigan Ann Arbor, MI United States; 2 Medical School University of Michigan Ann Arbor, MI United States

**Keywords:** augmented reality, emergency medicine, education, telemedicine

## Abstract

**Background:**

Augmented reality is increasingly being investigated for its applications to medical specialties as well as in medical training. Currently, there is little information about its applicability to training and care delivery in the context of emergency medicine.

**Objective:**

The objective of this article is to review current literature related to augmented reality applicable to emergency medicine and its training.

**Methods:**

Through a scoping review utilizing Scopus, MEDLINE, and Embase databases for article searches, we identified articles involving augmented reality that directly involved emergency medicine or was in an area of education or clinical care that could be potentially applied to emergency medicine.

**Results:**

A total of 24 articles were reviewed in detail and were categorized into three groups: user-environment interface, telemedicine and prehospital care, and education and training.

**Conclusions:**

Through analysis of the current literature across fields, we were able to demonstrate that augmented reality has utility and feasibility in clinical care delivery in patient care settings, in operating rooms and inpatient settings, and in education and training of emergency care providers. Additionally, we found that the use of augmented reality for care delivery over distances is feasible, suggesting a role in telehealth. Our results from the review of the literature in emergency medicine and other specialties reveal that further research into the uses of augmented reality will have a substantial role in changing how emergency medicine as a specialty will deliver care and provide education and training.

## Introduction

Since its inception as a specialty, emergency medicine has continually adapted and evolved in the way patient care is delivered as well as in training emergency medicine providers. From the adaptation and utilization of point-of-care ultrasound for procedures and clinical decision making to the methods in which critical care is delivered to patients—both in the prehospital setting and within the emergency department—emergency medical care has leveraged technological advances to improve outcomes for patients [1-3]. Similarly, emergency medicine has been an early adopter for a variety of technology-based education tools, such as simulation and free open-access medical education [4,5].

Augmented reality and virtual reality are two exciting, closely related, but fundamentally different emerging technologies. The central difference between the two technologies is that virtual reality is completely immersive: the headsets must, by necessity, block out the external world. However, augmented reality, by design, maintains the user’s connections with the real world. Augmented reality synthesizes the virtual and real; like virtual reality, an augmented reality experience typically involves a headset through which you can view a physical reality that has been augmented or supplemented by computer-generated sensory input such as sound, video, graphics, or GPS data [6]. Both augmented reality and virtual reality are expected to generate US $90 billion in revenue by 2022 [7]. Augmented reality in particular has substantial potential to provide powerful, contextual, and situated learning experiences as well as construct new understanding based upon the user’s interactions with virtual objects, which bring underlying data to life. With various augmented reality technologies, one has the ability to perform a wide variety of tasks, including displaying and manipulating information within one’s field of view, mapping virtual images to real objects, and video-conferencing [8,9].

Although there have been studies suggesting that there is significant potential for augmented reality in health care, including medical education and surgical subspecialties, at this time there has not been a review of the potential uses for augmented reality in the context of emergency medicine [10,11]. The aim of our paper was to review the medical literature as well as literature in other fields to determine the application of augmented reality in emergency care.

## Methods

We conducted a scoping review of both medical and nonmedical journals examining the current use of augmented reality with the assistance of a health sciences informationist (BS).

**Figure 1:**
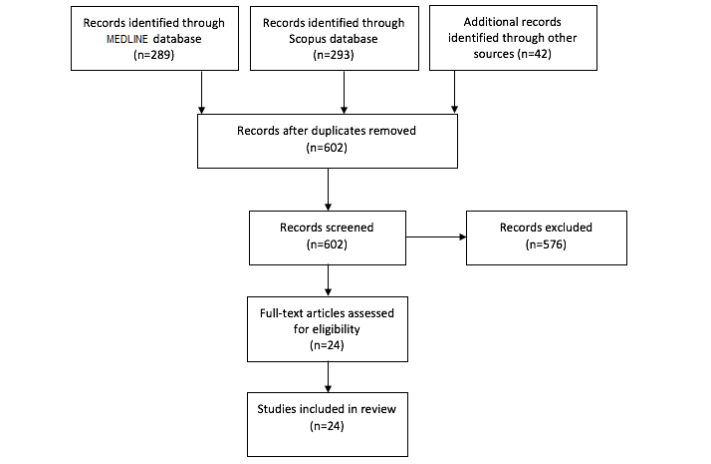
Preferred Reporting Items for Systematic Reviews and Meta-Analyses (PRISMA) flowchart of search strategy.

We elected to perform a scoping review because this area of research is still in its infancy and we therefore sought to quantify the number of articles with potential relevance to emergency medicine. The majority of articles were identified using the Scopus and MEDLINE databases, as well as Embase. We additionally performed a search for relevant articles within the grey literature; however, no records were identified. Following standard Preferred Reporting Items for Systematic Reviews and Meta-Analyses (PRISMA) guidelines for study selection, two authors (BWM and MK) evaluated the articles using title and abstract analysis for relevance to emergency medicine and its subspecialties, as detailed in Figure 1 [12]. A total of 20 articles were reviewed by both authors independently to assess interrater reliability, and it was found that there was complete agreement in review. The remaining articles were divided by the two authors evenly and reviewed for relevance. Any articles that were of questionable relevance were reviewed by the two authors independently to come to a consensus. If the authors were split, a third party (PM) was available as an additional reviewer.

Articles were defined as applicable to emergency medicine if there was relevance to a practice within the field of emergency medicine (ie, procedures within the scope of practice of an emergency medicine physician) or its subspecialties (ie, ultrasound or prehospital medicine); if the method in which augmented reality was studied could be applied to emergency medicine (ie, use of an augmented reality headset or novel functionality); and if there was potential educational or teaching value as it relates to emergency medicine. Articles that were excluded from this study were those that were not directly relevant to emergency medicine, were not studying augmented reality, or were not primarily published in the English language.

A total of 24 studies were identified as relevant to both augmented reality and emergency medicine. One study was noted to be directly related to emergency medicine. The remaining studies were felt to have topics that were applicable to emergency medicine or its subspecialties. Articles were accessed using the University of Michigan library system.

## Results

### Overview

A total of 24 articles were selected for full-article review. The main themes within the articles that were found to be relevant to emergency medicine included user-environment interfaces, telemedicine and prehospital care, and education and training. Some articles were found to relate to more than one primary theme. Due to the fact that there were a minimal number of articles directly involving the field of emergency medicine, the majority of the articles reviewed here are from other specialties that could have direct relatability to emergency medicine. Table 1 lists the categories of articles as they relate to emergency medicine [13-36].

### User-Environment Interface

Out of 24 articles, 6 (25%) evaluated the interactions between the user and environment. Articles were focused on topics ranging from augmented realism through haptic feedback and anatomic overlaying to usability without interference or distraction and assistance with clinical management.

Siebert et al utilized headsets with the Pediatric Advanced Life Support (PALS) algorithm for defibrillation during a mock code. It was found that there was no increase in time to initial defibrillation and there were fewer errors associated with dosages of defibrillation and medications in those using augmented reality [13]. Usability of the headsets was not discussed in this article. However, in a feasibility trial using Google Glass, Chaballout et al noted concerns regarding distraction through multiple images, overheating of hardware, and difficulties with establishing an Internet connection [14].

Rochlen et al evaluated the feasibility of augmented reality with headsets creating an anatomical layout of an internal jugular vein and carotid artery over a manikin for central venous catheterization insertion [15]. The authors found that, overall, participants found the use of augmented reality easy and enjoyable and found that it did not interfere with the procedural technique.

Abhari et al hypothesized that incorporation of augmented reality into surgical planning would result in improved task performance during resection of neurosurgical tumors. By utilizing a virtual image of the brain overlaying a phantom head, trainees were able to decrease cognitive load in planning a resection and focus more on appropriate technique and surgical maneuvers [16]. Performed in a training environment, this is a first step toward application of augmented reality of procedural tasks in a clinical setting.

Dickey et al utilized a headset with just-in-time training and instruction prior to performing a urologic procedure [17]. Additionally, during the actual procedure, an attending that was not present in the room communicated via headset and video-conferencing and was able to use a cursor to highlight important points within the procedure. Trainees and faculty members in this study felt that the system had educational value, was easy to navigate, and was not distracting. Similarly, Andersen et al describe a system in which orthopedic attendings can directly annotate onto the field of view by using a transparent display on a podium, thereby reducing shifts of focus during a procedure [18].

**Table 1:** Categorized search results as related to domains of emergency medicine.

Titles of studies in each emergency medicine domain		Journal or conference	First author, year published
**User-environment interface**			
	Adherence to AHA^a^ guidelines when adapted for augmented reality glasses for assisted pediatric cardiopulmonary resuscitation: A randomized controlled trial	Journal of Medical Internet Research (JMIR)	Siebert J, 2017 [13]
	Feasibility of augmented reality in clinical simulations: Using Google Glass with manikins	JMIR Medical Education	Chaballout B, 2016 [14]
	First-person point-of-view augmented reality for central line insertion training: A usability and feasibility study	Simulation in Healthcare	Rochlen L^b^, 2017 [15]
	Training for planning tumour resection: Augmented reality and human factors	IEEE^c^ Transactions on Biomedical Engineering	Abhari K, 2015 [16]
	Augmented reality assisted surgery: A urologic training tool	Asian Journal of Andrology	Dickey R^d^, 2016 [17]
	Avoiding focus shifts in surgical telementoring using an augmented reality transparent display	Studies in Health Technology and Informatics	Andersen D, 2016 [18]
**Telemedicine and prehospital care**			
	Telemedicine supported by augmented reality: An interactive guide for untrained people in performing an ECG^e^ test	Biomedical Engineering Online	Bifulco P, 2014 [19]
	Augmented reality as a telemedicine platform for remote procedural training	Sensors	Wang S, 2017 [20]
	Virtual interactive presence in global surgical education: International collaboration through augmented reality	World Neurosurgery	Davis MC, 2016 [21]
	Telementoring: Use of augmented reality in orthopaedic education: AAOS^f^ exhibit selection	The Journal of Bone & Joint Surgery	Ponce BA, 2014 [22]
	Seven years of clinical experience with teleconsultation in craniomaxillofacial surgery	Journal of Oral and Maxillofacial Surgery	Ewers R, 2005 [23]
	Augmented reality assisted surgery: A urologic training tool	Asian Journal of Andrology	Dickey R^d^, 2016 [16]
	Disaster medicine through Google Glass	European Journal of Emergency Medicine	Carenzo L, 2015 [24]
	Technical support by Smart Glasses during a mass casualty incident: A randomized controlled simulation trial on technically assisted triage and telemedical app use in disaster medicine	Journal of Medical Internet Research (JMIR)	Follmann A, 2019 [25]
**Education and training**			
	Design of mobile augmented reality in health care education: A theory-driven framework	JMIR Medical Education	Zhu E, 2015 [26]
	Systematic review on the effectiveness of augmented reality applications in medical training	Surgical Endoscopy	Barsom EZ, 2016 [27]
	Personalized augmented reality for anatomy education	Clinical Anatomy	Ma M, 2016 [28]
	Augmented reality for anatomical education	Journal of Visual Communication in Medicine	Thomas RG, 2010 [29]
	The effectiveness of virtual and augmented reality in health sciences and medical anatomy	Anatomical Sciences Education	Moro C, 2017 [30]
	Modification of commercial force feedback hardware for needle insertion simulation	Studies in Health Technology and Informatics	Coles TR, 2011 [31]
	Augmented reality for teaching endotracheal intubation: MR^g^ imaging to create anatomically correct models	Annual AMIA^h^ Symposium Proceedings	Kerner KF, 2003 [32]
	First-person point-of-view augmented reality for central line insertion training: A usability and feasibility study	Simulation in Healthcare	Rochlen L^b^, 2017 [15]
	Advanced training methods using an augmented reality ultrasound simulator	IEEE 2009 International Symposium on Mixed and Augmented Reality	Blum T, 2009 [33]
	An augmented reality simulator for ultrasound guided needle placement training	Medical & Biological Engineering & Computing	Magee D, 2007 [34]
	Piloting augmented reality technology to enhance realism in clinical simulation	Computers, Informatics, Nursing	Vaughn J, 2016 [35]
	An investigation of university students' collaborative inquiry learning behaviors in an augmented reality simulation and a traditional simulation	Journal of Science Education and Technology	Wang H-Y, 2014 [36]

^a^AHA: American Heart Association.

^b^Cross-listed in User-environment interface and Education and training.

^c^IEEE: Institute of Electrical and Electronics Engineers.

^d^Cross-listed in User-environment interface and Telemedicine and prehospital.

^e^ECG: electrocardiogram.

^f^AAOS: American Academy of Orthopaedic Surgeons.

^g^MR: magnetic resonance.

^h^AMIA: American Medical Informatics Association.

### Telemedicine and Prehospital Care

There were 8 out of 24 articles (33%) focusing on the utility of augmented reality in the prehospital environment or through remote locations. Articles in this section focused on feasibility of providing instruction from a remote location, accuracy, and timeliness of feedback provided. Additionally, this section looks at articles that utilize augmented reality for prehospital care and triage in disaster management.

Augmented reality-supported telemedicine was used by both Bifulco et al and Wang et al to train novices in procedures [19,20]. Bifulco et al demonstrated that it was feasible to use augmented reality to assist with training novices to correctly perform an electrocardiogram (ECG) with minimal errors through the use of cameras, headsets, and annotations. Similarly, Wang et al used the Microsoft HoloLens to remotely instruct novices on point-of-care ultrasound. A facilitator guided learners through remote image capture of hand positions, providing instruction on complex maneuvers. Overall, results from this study demonstrated feasibility in an augmented reality platform for telemedicine as well as feasibility of the HoloLens as a headset.

Davis et al used an iPad-based virtual and augmented reality-based tool to communicate from Birmingham, Alabama, to Ho Chi Minh City, Vietnam, and provide both visual and verbal cues to assist with neurosurgical procedures [21]. The average length of delay in communication was 237 milliseconds and there was no disruption in the quality of the image transmitted. Similarly, Ponce et al used the same tool to demonstrate effectiveness with orthopedic residents performing arthroscopic shoulder surgery [22]. In this study, both residents and attendings felt that the use of augmented reality was educational, was easy to use, and provided immediate feedback to the trainee without causing a significant lag or interference with the procedure. Ewers et al assisted in 50 procedures over a 7-year period using augmented reality, noting that 48 of 60 (80%) transmission attempts were successful with good image quality [23]. As mentioned above, Dickey et al used remote location attendings to provide assistance and guidance for residents performing a urologic procedure [17].

Carenzo et al demonstrated an application for augmented reality in disaster triage using Google Glass. Through the development of an app based upon geomarked quick-response (QR) codes, electronic triaging of disaster patients was performed in a mock triage event. Using hands-free technology and Google Glass as a QR scanner, emergency medical services providers were directed to appropriate patients in an efficient manner [24]. Follmann et al compared traditional triaging during a simulated mass casualty event to the use of Smart Glasses through both an interactive triage algorithm app and telemedical support. The study found that while traditional triaging was faster, triaging using both an interactive Smart Glasses app and a physician with triage experience available by telemedicine was significantly more accurate [25].

### Education and Training

Out of 24 articles, 12 (50%) regarding augmented reality with relation to emergency medicine had a focus on education and training. Several of the articles were systematic reviews of the use of augmented reality in medical education as well as conceptual frameworks that can be applied to multiple specialties. Education articles focused on both procedural training as well as clinical decision making in a simulated environment.

Zhu et al proposed a framework when developing augmented reality applications, based upon three different learning theories: situated learning, experiential learning, and transformative learning. By incorporating augmented reality into a realistic learning environment that is insulated from the high-stakes patient care location, the authors propose that the learning that takes place in an augmented reality-assisted environment can inform real practice, while allowing for reflection through experiences [26]. In considering this framework for development of augmented reality applications, there is an emphasis on developing multiple learning modalities within an environment.

Barsom et al performed a systematic review of augmented reality systems that exist that have been validated within a training environment. With specific relation to emergency medicine, two separate echocardiography augmented reality applications were reviewed: one that was felt to be highly realistic, suggesting face validity, and the other determined to have construct validity as determined by different levels of competency for experts, intermediate learners, and novices [27].

Several author groups have looked at the use of augmented reality in anatomical education. Ma et al utilized computed tomography (CT)-developed imaging overlaid over learners for educational benefit. Learners then stood in front of a front-facing video camera, overlaying anatomical images on their bodies [28]. Users found this to be an enjoyable way to relate anatomy to a real situation. Thomas et al used an augmented reality model for developing 3D brains for anatomical study, while also adding a tactile component for interactive learning [29]. Their model allowed for annotation and manipulation in a 3D environment without relying on conventional dissection. Moro et al compared virtual reality, augmented reality, and tablet-based methods for teaching skull anatomy [30]. The study found that virtual and augmented reality were equally effective in teaching anatomy but promoted increased engagement and learner immersion due to the tactile and 3D nature of these learning modes. Virtual reality-based learning was associated with increased episodes of dizziness compared to the other two modalities.

One of the most common utilities for augmented reality currently being investigated is procedural learning. Coles et al used a simplified hydraulic pump to simulate a femoral pulse for needle placement in an interventional radiology. Using augmented reality, the authors simulated the clinical environment as well as the actual needle insertion [31]. The study found that learners felt the model was realistic and was educational. Kerner et al used magnetic resonance-based images to create anatomically correct airways to teach learners appropriate technique for endotracheal intubation [32]. By overlaying images using augmented reality, facilitators were able to demonstrate the changes in airway patency with changes in positioning, such as the sniffing position, extension, and hyperflexion. Rochlen et al used an anatomical overlay to teach learners appropriate technique for central venous catheterization [15].

In addition to the above procedural skills, augmented reality has been used to simulate ultrasound training as well as ultrasound-guided procedures. Blum et al used CT images with a phantom ultrasound simulator to create ultrasound images from CT slices [33]. Sensors placed on the probe, as well as the phantom manikin, interact to simulate ultrasound images from the existing CT scan. Magee et al used augmented reality to simulate ultrasound images and anatomy for interventional radiology procedures [34]. Participants felt that there was face validity in the simulated procedure, in that the augmented reality needle placement accurately replicated the look of the true procedure. It is noted in this article that there was not a mechanism for tactile feedback and the procedure was therefore not considered to feel realistic by participants.

Vaughn et al piloted augmented reality technology using Google Glass with nursing students to add additional realism to a simulated manikin scenario [35]. Learners felt that a simulated patient video provided an added level of realism to the scenario, giving them confidence when they successfully completed the scenario. Wang et al compared students undergoing traditional simulation scenarios with students participating in augmented reality simulation scenarios. Students manipulated virtual 3D cubes to teach them about physics concepts [36]. The behaviors and interactions of the groups were compared, and it was found that students participating in augmented reality simulation scenarios were more supportive of each other in a collaborative environment, though the sample size was small and significance was not reached.

## Discussion

### Principal Findings

Our scoping review revealed that a majority of studies that have been conducted can be categorized into one of three distinct themes: *user-environment interfaces*, *telemedicine and prehospital care*, and *medical education and training*. Furthermore, our analysis revealed that most of the studies were observational in nature and were conducted on small cohorts of participants. Despite these limitations, we believe that augmented reality has a substantial potential to impact emergency care but would need to be investigated in a more robust manner.

The studies cited above have shown that augmented reality can provide useful user-environmental interfaces in a health care context. Emergency department care demands real-time decision making that requires analysis of objective and subjective data that currently is displayed on computer screens and monitors that divert provider attention away from patients. Live vital sign information along with laboratory data and imaging can all be displayed on an augmented reality platform that limits provider distraction from his or her patient. Furthermore, in the same way that augmented reality has been used in surgical settings in the context of procedures and procedural planning, augmented reality can be used during live procedures in the emergency department. For example, ultrasound imaging can be added to a visual interface that limits providers from looking away from their patient and their needle and devices [15,33]. Expert guidance during procedures and telemedicine can also be delivered via such a platform [17,18,25].

### Future Directions

Augmented reality has potential in both the prehospital and telemedicine settings. Similar to the example above, prehospital providers can benefit from expert guidance with visual instructions relayed to their augmented reality devices. Such devices can also provide visual and audio streams for emergency departments who are expecting the arrival of critical patients to help guide their initial resuscitation. Augmented reality can provide an avenue through which rural and international emergency departments can be given guidance on specialized care and procedures. Similarly, augmented reality is a modality that has the potential to revolutionize medical training and education. Augmented reality grants the potential to create hyper-realistic simulations that can give learners a safe environment in which to learn various procedures and skills.

As an emerging technology, augmented reality has substantial potential to enhance quality of care, improve access, and reduce errors especially when contextualized to acute care. To date, there are no studies that evaluate the effect of augmented reality on patient safety in real time. It will be important in developing this technology to evaluate not only its impact on skill acquisition and telemedicine, but to evaluate the use of augmented reality on patient safety and clinical outcomes.

One of the major advantages of augmented reality is that, unlike other computer interfaces, it analyzes and displays information within an individual’s field of view, thus allowing information to be utilized in real time. In emergency medicine, appropriate delivery of care often requires quick and easy access to pertinent data that is currently delivered via monitors and computer screens. This can be distracting and lead to inefficiencies which augmented reality can minimize. Additionally, during surgery and other procedures, augmented reality can provide a modality through which information can be delivered to providers in a novel way. Medical education can also benefit from augmented reality, as simulation and basic anatomic and physiological concepts can be displayed in ways that 2D screens cannot achieve.

Prior to augmented reality, the primary modality of interfacing with such information has been through different types of screens and monitors. Augmented reality requires considerable computing and hardware capabilities, and therefore wide-scale development of augmented reality has been limited until recently. Augmented reality software and hardware are now being actively developed by major companies, such as Apple, Microsoft, Facebook, and Google, and is set to become much more ubiquitous in the near future [37].

Historically, medicine as a field has taken time to adapt emerging general-purpose technologies. Despite the widespread use of digital records in other fields, electronic medical records have only recently become mandated [38].

The success of augmented reality as a data interface for providers in the emergency department will be based upon a variety of factors. Cost, software development, and portability will all be crucial determining factors on whether or not augmented reality will find a place in the emergency department of the future. Devices such as the Microsoft HoloLens provide the functionality necessary for widespread application but may not be realistic to wear in the emergency department environment yet. Currently, companies such as Apple are investigating the use of augmented reality as an extension of their mobile phones [37]. As this technology advances, more studies will be needed to evaluate the different modalities for which augmented reality can be used. Most of these factors are dependent on advancements in the private sector. Future augmented reality devices will likely also have to be able to connect and interface with other medical devices, such as vital monitors, ultrasound machines, and emergency medical record systems, in order to be successful. This will require software standards to be developed. Finally, augmented reality will have to prove beneficial to patient outcomes, provider education, emergency department efficiency, and overall cost reduction to convince health care systems to invest in such technologies.

### Limitations

Our review did have limitations that are worth discussing. This review was limited to studies from two databases—MEDLINE and Scopus—and at this time there is no way to evaluate augmented reality applications that are being developed by private enterprise. Our initial aim was to find all studies that could be generalized to emergency medicine as a specialty and did not focus on one specific application within the field of emergency medicine. However, as augmented reality is a nascent technology, research involving it is limited, and a more specific clinical question would not have yielded enough papers to conduct such a review. Another limitation is that the included studies described small-scale implementations of augmented reality and use various different augmented reality platforms in their research. Lastly, the majority of these studies did not use objective data to evaluate augmented reality effectiveness. As augmented reality becomes more ubiquitous, we believe that these limitations will pose less of an issue.

In addition to the limitations described above, there are limitations to the current devices on the market. As this is a new technology, the wearables often have low battery life, less-than-ideal camera quality, and the potential for poor wireless connection. Furthermore, there is little information on usability in a health care setting with regard to activities such as sanitation and usability. Given that augmented reality is an emerging technology, the hand swipes and gestures may be cumbersome for health care providers, akin to when advanced-feature mobile phones were first introduced. As with any new technology, it can take time to develop the requisite technology that will suit the needs of health care. These limitations also stress the importance of quality future research looking at the feasibility of using augmented reality in novel settings such as the emergency department, especially as it relates to the devices and user interactions. Future studies should not only investigate this but compare it to current technology to determine its benefits.

### Conclusions

An important conclusion that can be drawn from this review is that any research involving augmented reality can be used to inform other applications across various disciplines and areas. We believe that augmented reality has a plethora of applications in emergency medicine and possesses the potential to revolutionize delivery of patient care and medical education. However, further research is needed and should focus on augmented reality’s effectiveness in tackling the more specific problems faced in medical settings. Though our review has focused primarily on the emergency department, many of these challenges may be relatable to other clinical environments in medicine. Our review found that although current research is sparse in augmented reality and its application for clinical care delivery and education, augmented reality has substantial potential to change the paradigm of care for acutely ill and injured patients and populations.

## Abbreviations

AAOS: American Academy of Orthopaedic Surgeons
AHA: American Heart Association
AMIA: American Medical Informatics Association
CT: computed tomography
ECG: electrocardiogram
IEEE: Institute of Electrical and Electronics Engineers
MR: magnetic resonance
PALS: Pediatric Advanced Life Support
PRISMA: Preferred Reporting Items for Systematic Reviews and Meta-Analyses
QR: quick response
